# Knowledge, attitude, and practice towards hepatitis B and C virus infection and associated factors among adults living at selected woredas in Gamo Zone, Southern Ethiopia: a cross-sectional study

**DOI:** 10.1186/s12889-024-18387-z

**Published:** 2024-04-09

**Authors:** Tamiru Chonka, Gesila Endashaw, Eshetu Zerihun, Bereket Beyene Shashamo

**Affiliations:** 1 School of Nursing, Department of Comprehensive Nursing, College of Medicine and Health Sciences, Arba Minch University, Arba Minch, Ethiopia; 2 School of Public Health, Department of Nutrition, College of Medicine and Health Sciences, Arba Minch University, Arba Minch, Ethiopia

**Keywords:** Hepatitis, Knowledge, Attitude, Practice, Adults, Southern ethiopia

## Abstract

**Background:**

Hepatitis is an inflammation of the liver tissue. It is one of the serious public health problems. Though an individuals’ knowledge, attitude, and practice level is very vital in order to ensure the control of its adverse health impacts, little is known regarding these issues in the community level. Therefore, this study was aimed to assess knowledge, attitude, and practice towards hepatitis B and C virus infection and associated factors among adults living at selected woredas in Gamo Zone, Southern Ethiopia.

**Methods:**

Community based cross-sectional study design was conducted among 633 adults living at selected woredas in Gamo Zone, Southern Ethiopia. Data were collected by pretested, well-structured questionnaire. The collected data were checked, coded and entered into Epi-data version 4.6.0.2 and were exported to SPSS version 25 for analyses. Bivariable and multivariable logistic regression were done to identify independent factors associated with knowledge, attitude, and practice towards hepatitis B and C virus infection.

**Results:**

According to this study, 366**(**58.1%), 95% CI: (54.23–61.96) of the participants had good knowledge. 381**(**60.5%), 95% CI: (56.65–64.30) of the participants had favourable attitude. 317**(**50.3%), 95% CI: (46.40–54.23) of the participants had good practice. From factor analysis, sex, number of sexual partners, sharing sharp material, and vaccination status were significantly associated with knowledge; residence, occupational status, income level, sharing sharp material, and vaccination status were significantly associated with attitude; and residence, occupational status, and vaccination status were identified to be significantly associated with practice towards Hepatitis B and C virus infection.

**Conclusion:**

Based on the study findings, it could be observed that good knowledge, favourable attitude, and good practice were indicated nearly above the half, only by half, and nearly above the half of the study participants respectively. Implementing actions that could increase awareness regarding limiting the number of sexual partner, not sharing sharp materials, and urbanization is recommended. Moreover, woreda administrators, and other related authorities should consider knowledge, attitude, and practice as an implementation area, and also it would be better to create an opportunities to promote vaccination practices.

**Supplementary Information:**

The online version contains supplementary material available at 10.1186/s12889-024-18387-z.

## Background

Hepatitis is an inflammation of the liver tissue [1]. Heavy alcohol use, toxins, some medications, and certain medical conditions can cause hepatitis. However, hepatitis is often caused by a virus [1]. There are five types of hepatitis (A, B, C, D, E) there are also other types of non-classified or with non-obvious link with this disease, such as hepatitis virus G 2 [2]. Although there are five distinct types of viral hepatitis, chronic hepatitis B and C cause 95% of hepatitis-related sickness and untimely deaths [3].

Currently, there is an HBV immunisation programme in several countries of the world [4]. In Ethiopia, the Expanded Programme for Immunisation (EPI) was launched in 2007 and includes the pentavalent [DPT-HiB-HepB] vaccine, which includes the HBV immunisation. EPI regimens state that the HBV vaccine is administered six, ten, and fourteen weeks following delivery [5]. Its coverage has grown to 90% and is still growing. Studies, however, reveal that despite widespread vaccination, people are still susceptible to HBV infection [6].

Globally, 296 million people are living with hepatitis B and 58 million people are living with hepatitis C [7]. In 2019, its estimates revealed that 1.1 million deaths occurred due to these infections and their adverse health effects include liver cancer, cirrhosis, and other conditions caused by chronic viral hepatitis [7]. It is one of serious public health problem. About 15–20% of people who get infected by the hepatitis B develop chronic liver disease, including cirrhosis, liver failure, or liver cancer and more than 50% of people who get infected with the hepatitis C virus develop a chronic infection [8]. Also, 5-25% of people with chronic hepatitis C develop cirrhosis over 10–20 years [8].

By assessing resident’s knowledge, attitude and their regular practices towards hepatitis, concerned stakeholders can take actions to help improve the resident’s lifestyle by improving their awareness level which will positively lead to their best beliefs towards hepatitis and ultimately better practices [9]. Additionally, research on knowledge, attitude, and practice (KAP) on hepatitis-related data was encouraged since it will undoubtedly aid in the development and implementation of effective preventative and treatment plans [10]. Therefore, this study was proposed to assess knowledge, attitude and practice (KAP) levels towards hepatitis B and C infection among adults in the community base.

In Ethiopia, according to a comprehensive literature review which was carried out from five decades (1968–2015), the overall pooled prevalence of hepatitis B virus (HBV) was 7.4% and the overall pooled prevalence of anti-hepatitis C virus antibody (anti-HCV) was 3.1% [11]. In 2014, sero-prevalence of hepatitis B and C virus infections among pregnant women were found to be 4.4 and 0.26%, respectively [12]. In 2018, the overall prevalence of HBsAg and anti-HCV among refugees was 7.3% (33/453) and 2.0% (9/453) [13]. Within the same year, a study conducted in South Omo zone has revealed that the sero-prevalence for hepatitis B (HBV) infection was 8.0% and the sero-prevalence for hepatitis C (HCV) infection was 1.9% [14]. From this, we can see that it is an existing problem in our setting.

Regarding the knowledge, attitude and practice towards hepatitis infection, different studies have revealed that the magnitude of poor knowledge, unfavourable attitude and poor practice are an existing public health problem. In Malaysia, the magnitude of poor knowledge towards hepatitis B was 63% among households [9]. In Ethiopia, the magnitude of poor knowledge towards hepatitis B virus infection was 48% among health science students [15]. In China, the magnitude of unfavourable attitude towards hepatitis infection was 16.7% among pregnant women [16]. In Sudan, it was 13.6% among nurses and midwives [17]. In Ethiopia, it was 46.7 among pregnant women [18]. In Sudan, the magnitude of poor practice towards hepatitis infection was, 34.5% among nurses and midwives [17]. In Ethiopia, it was 57.4% among health care workers [19].

In 2016, WHO’s World Health Assembly (WHA) called for global elimination of viral hepatitis by 2030 [20]. And set global targets of achieving 90% reduction in new cases of hepatitis B and hepatitis C, a 65% reduction in deaths from hepatitis B and hepatitis C, and treatment of 80% of people living with these infections [20]. In order to achieve all of these goals, accurate public awareness is very vital and that is why knowledge, attitude and practice (KAP) studies are so important for ensuring adequate preventive measures in the community.

Though studies were conducted in different parts of the world, we have noticed gaps in the literature regarding population, implications, and setting variances, despite the fact that investigations were done in various parts of the world.

Coming to the Ethiopian setting, even though the concern is given to knowledge, attitude and practice towards hepatitis, most of the findings were from the perspectives of health care providers [21, 22], pregnant mothers visiting medical care institution [18, 23], and from medical and health sciences students [15, 24], and these findings can’t be generalized to the community level. Hence, residents in the community have a big chance of acquiring hepatitis infection, the findings from community or non-health care professional side are very vital in order to ensure prevention of hepatitis to the adequate level. Furthermore, no prior research in the study area has been conducted with objectives comparable to those of this study. Therefore, this study addressed these gaps by assessing knowledge, attitude and practice towards hepatitis B and C virus infection and associated factors among adults living at selected woredas in Gamo Zone, Southern Ethiopia.

## Methods

### Study area and period

This study was conducted in selected woredas of Gamo Zone, Southern Ethiopia from June 1 to 30, 2022. Gamo zone is located in southern nation, nationalities and people’s regional state, southern Ethiopia. Its capital is Arba Minch town. It founds about 500 km south of Addis Ababa, at an elevation of 1285 m above sea level. It is the largest town in Gamo Zone and the second town in SNNPR next to Hawassa. This study was conducted at selected woredas of Gamo zone; Namely; Kucha, Daramalo and Kamba woreda.

### Study design

Community based cross sectional study was employed.

### Population

#### Source population

All adult population living at Kucha, Daramalo, and Kamba woredas. The estimated total population was 104,429, 96,936, and 120,979 for Kucha, Daramalo, and Kamba woreda, respectively.

### Study population

All adult population living at selected kebeles in Kucha, Daramalo and Kamba woredas during the data collection period and fulfil the inclusion criteria.

### Inclusion and exclusion criteria

The participants in this research had to be at least 18 years old and have lived in the chosen kebele for at least six months. Participants who were gravely sick and unable of responding at the time the data were collected were not included in the research.

### Sample size determination and sampling procedure

Sample size was calculated using single population proportion formula with the assumptions of Confidence level = 95%, Critical value Zα/2 = 1.96, Degree of precision d = 0.05, the proportion (p) = 50% because to the level of our literature review, there was no study done in the same setting and population as this study concerning knowledge, attitude and practice of adult residents towards hepatitis infection. Hence we used multistage sampling, we have used a design effect of 1.5 and nonresponse rate of 10% was added on the final sample size. Based on this, the final sample size of this study was 633. To obtain this amount of sample, multistage sampling technique was used. For that, three woredas (i.e. Kucha, Daramalo and Kamba) are selected purposively. According to zonal health department, there were 81 kebeles in these woredas (i.e., 24 in Daramalo, 26 in Kucha, and 31 in Kamba). From the total number of kebeles found in each woreda, 25% of them (i.e. 6 from daramalo. 6 from kucha and 8 from kamba) were taken randomly. Next to that, the sample size was distributed proportionally to each kebele based on the total number of households found in each of them. Then, a list of households was obtained from the respective kebele administration offices and used as a sampling frame, and then households were selected using systematic sampling technique. For each selected kebele, the sampling interval “k” was determined (k = N/n) and the first interviewed household was identified using a lottery method among the households in the first sampling interval “K1”. Finally, from each selected households one participant was recruited randomly using lottery method if there were more than 1 eligible person.

### Data collection tool, data collectors, and procedure

Data were collected by using well-structured questionnaire. The questionnaire contains written consents, items for assessing socio-demographic variables, health related behaviours, knowledge, attitude and practice related questions. It was adapted from related previous peer-reviewed literatures. Twenty data collectors and twenty supervisors were recruited to handle the data collection process. The study participants were selected from individuals aging 18 years and above, and fulfil inclusion criteria. Adults reporting illness and immigrants from other area were excluded. The data collectors collected the data through a pretested well-structured questionnaire. They informed the adults about all details of the research. The participants were encouraged to feel free and were told that the confidentiality of their responses will be assured and no information will be shared with third parties, and their name will be not written on the questionnaire. After this, adults who were willing to participate and those who signed the informed, voluntary written consent document were interviewed in their home.

### Study variables

Knowledge, attitude and practice towards hepatitis B and C infection were dependent variables and socio-demographic characteristics and health related characteristics were independent variables in this study.

### Operational definitions

#### Knowledge

awareness about the disease, ways of transmission, and prevention.

#### Good knowledge

refers for those study participants who scored point greater than or equal the mean for knowledge related questions. Knowledge-related questions comprise 10 items, and each question was labelled with good or poor knowledge. The good response was coded as 1, the poor response was coded as 0, and the total sum score ranged from 0 to 10. Then, the mean value was computed, and knowledge was considered good when the score was mean and above the mean value and poor when the score was below the mean.

#### Attitude

is the perception of participants having about a learned predisposition to think, feel and act in a particular way towards a given situation.

#### Favourable attitude

Refers to those study participants who scored point greater than or equal to the mean for attitude related questions. Attitude-related questions comprise 5 items, and each question was labelled with a favourable or unfavourable attitude. The favourable response was coded as 1, the unfavourable response was coded as 0, and the total sum score ranged from 0 to 5. Then, a mean value was computed, and attitude was considered favourable when the score was mean and above the mean value and considered unfavourable when the score was below the mean.

#### Practice

is the application of prevention practices.

#### Good practice

refers to those study participants who scored point greater than or equal to the mean to practice related questions. Practice-related questions comprise 5 items, and each question was labelled with good or poor practice. The good response was coded as 1, the poor response was coded as 0, and the total sum score ranged from 0 to 5. Then, the mean value was computed, and practice was considered good when the score was mean and above the mean value and considered poor when the score was below the mean.

### Data quality control

For ensuring data quality, the questionnaire was initially prepared in English and then translated in to local language by experts who have good skill of the two languages then translated back to English by different person to ensure consistency. One day training was given for data collectors and supervisors on objectives of the study, questionnaires, and ways of conducting data collection. Pre-test was conducted on 5% of the sample in Mirab Abaya woreda a week before the actual data collection. The data collection process was followed on daily base by the supervisors and investigators. The collected data were checked its completeness and consistency every day by the supervisors and investigators.

### Data processing and analyses

The collected data were coded, and entered into Epi data version 4.6.0.2. Then, the data were exported to SPSS window version 25 for further analyses. Descriptive analyses such as: simple frequencies, measures of central tendency, and measures of variability were used to describe the characteristics of the participants. Bivariable analyses was done and independent variables that yield p-value of ≤ 0.25 were included in the multivariable analyses to control all possible confounders and to detect true predictors of knowledge, attitude and practice towards hepatitis and C infection among adults. Multi-collinearity was checked. Normality test was conducted. An adjusted odds ratio with 95% CI was estimated to identify the factors associated with knowledge, attitude and practice towards hepatitis. The level of statistical significance was declared at p-value ≤ 0.05.

## Results

### Socio-demographic characteristics of the study participants

Out of 633 expected participants, 630 had participated in this study making a response rate of 99.53%. Among the respondents, 403(64.0%) were male. The mean age was 32.06 (SD ± 7.36) years and nearly half of the respondents, 291(46.2%) lies between 30 and 41 age group. The mean average monthly income was 2087.2 (SD ± 2444.84) and three fourth of the respondents, 475(75.4%) had monthly income less than 2000 in ETB. Also more than half of them, 406(64.4%) were married. Regarding their educational status, the largest proportion of the participants, 344(54.6%) didn’t attend a formal education (Table 1).

**Table 1:** Socio-demographic characteristics of the study participants (*n* = 630)

Variables	Category	Frequency(N)	Percent(%)
Sex	Male	403	64.0
	Female	227	36.0
Age in years	18–29 years	222	35.2
	30–41 years	291	46.2
	> 41 years	117	18.6
Residence	Urban	82	13.0
	Rural	548	87.0
Religion	Orthodox	255	40.5
	Protestant	340	54.0
	Others	35	5.6
Marital status	Single	159	25.2
	Married	406	64.4
	Widowed/Divorced	65	10.4
Educational status	No formal education	344	54.6
	Primary school	115	18.3
	Secondary school	94	14.9
	College and above	77	12.2
Occupational status	Government employed	60	9.5
	Day laborer	40	6.3
	Merchant	57	9.0
	Student	55	8.7
	Farmer	418	66.3
Monthly income in ETB	< 2000	475	75.4
	2000–4000	108	17.1
	> 4000	47	7.5

***Notes*** Others = Catholic and Muslim

### Health-related characteristics of the study participants

Among the participants, 569(90.3%) of the participants don’t had the multiple sexual partner. The greater proportion of the participants, 444(70.5%) didn’t share sharp materials with others. This study found that 433(68.7%) of the participants do not had a tattooing history. Three-fourths of the participants, 475 (75.4%), didn’t have the hepatitis vaccination (Table 2). About 222 (35.2%) of the respondents lie in the 18–29 age group (Table 1), which is approximately the young adult age group. And this has its own implications for the chronicity of the hepatitis in case these young people get infected.

**Table 2:** Health-related characteristics of the study participants (*n* = 630)

Variables	Category	Frequency (N)	Percent (%)
Multiple sexual partners	Yes	61	9.7
	No	569	90.3
Share sharp materials with others	Yes	186	29.5
	No	444	70.5
Have a tattooing history	Yes	197	31.3
	No	433	68.7
Vaccination	Yes	155	24.6
	No	475	75.4

### Participants’ knowledge towards hepatitis B and C virus infection

Out of the 630 participants, 301(47.8%) of the participants didn’t know Hepatitis can be transmitted through unsafe sexual intercourse. While 329(50.8%) of them responds they can get Hepatitis through body fluids contact. Moreover, 332(52.7%) of the participants responds Hepatitis can affect liver (Table 3).

**Table 3:** Knowledge of the participants towards hepatitis B and C virus infection (*n* = 630)

Knowledge on Hepatitis Infection	Response			
	Yes		No	
	Frequency(N)	Percent (%)	Frequency(N)	Percent(%)
Have you ever heard about hepatitis infection?	525	83.3	105	16.7
Can Hepatitis affect any age groups?	401	63.7	229	36.3
Is hepatitis transmitted through unsafe sex?	329	52.2	301	47.8
Can you get hepatitis infection through body fluid contact?	320	50.8	310	49.2
Can Hepatitis transmitted by instruments used for shaving and hair cutting?	311	49.4	319	50.6
Can Hepatitis be transmitted from mother to child?	315	50.0	315	50.0
Is Hepatitis curable/ treatable?	314	49.8	316	50.2
Can Hepatitis be prevented?	351	55.7	279	44.3
Is vaccination available for Hepatitis?	342	54.3	288	45.7
Does hepatitis cause liver cancer?	332	52.7	298	47.3

### The level of knowledge towards hepatitis B and C virus infection

The overall level of knowledge was computed from the score for knowledge related questions and the score of mean and above were considered as there is a good knowledge and the score of below mean were considered as there is poor knowledge. The mean score was obtained from the total sum score of correct/good response for total knowledge–related questions.

**Fig. 1:**
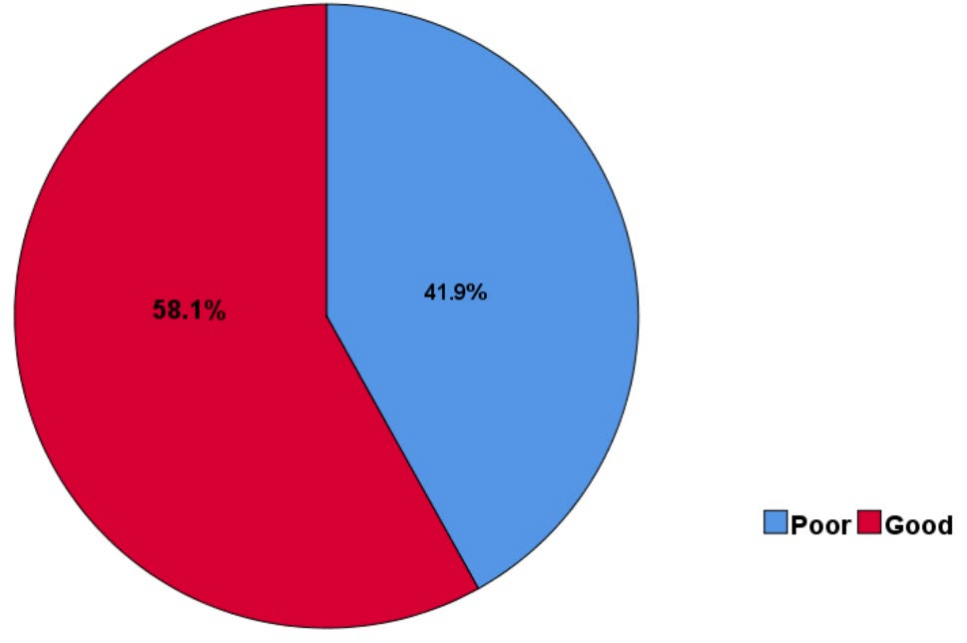
The level of knowledge towards hepatitis B and C infection (*n* = 630)

According to this, 366**(**58.1%), 95% CI: (54.23–61.96) of the participants had good knowledge (Fig. 1).

### Participants’ attitude towards hepatitis B and C virus infection

According to the findings of this study, 342(54.3%) of the participants don’t think that they can get Hepatitis. 395(62.7%) of them think that Hepatitis is a serious public health problem. Whereas, 392(62.2%) of the participants think that taking Hepatitis vaccine is necessary (Table 4).

**Table 4:** Attitude of the participants towards hepatitis B and C virus infection (*n* = 630)

Attitude towards Hepatitis infection	Response			
	Yes		No	
	Frequency(N)	Percent(%)	Frequency(N)	Percent(%)
Do you think you can get Hepatitis?	288	45.7	342	54.3
Do you perceive that hepatitis can be transmitted through food?	265	42.1	365	57.9
Do you think hepatitis infection is a curable disease?	347	55.1	283	44.9
Do you think that hepatitis is a serious public health problem?	395	62.7	235	37.3
Do you think that taking hepatitis vaccine is necessary?	392	62.2	238	37.8

### The level of attitude towards hepatitis B and C virus infection

The overall level of attitude was computed from the score for attitude related questions and the score of mean and above were considered as there is a favourable attitude and the score of below mean were considered as there is unfavourable attitude. The mean score was obtained from the total sum score of favourable response for total attitude–related questions.

**Fig. 2:**
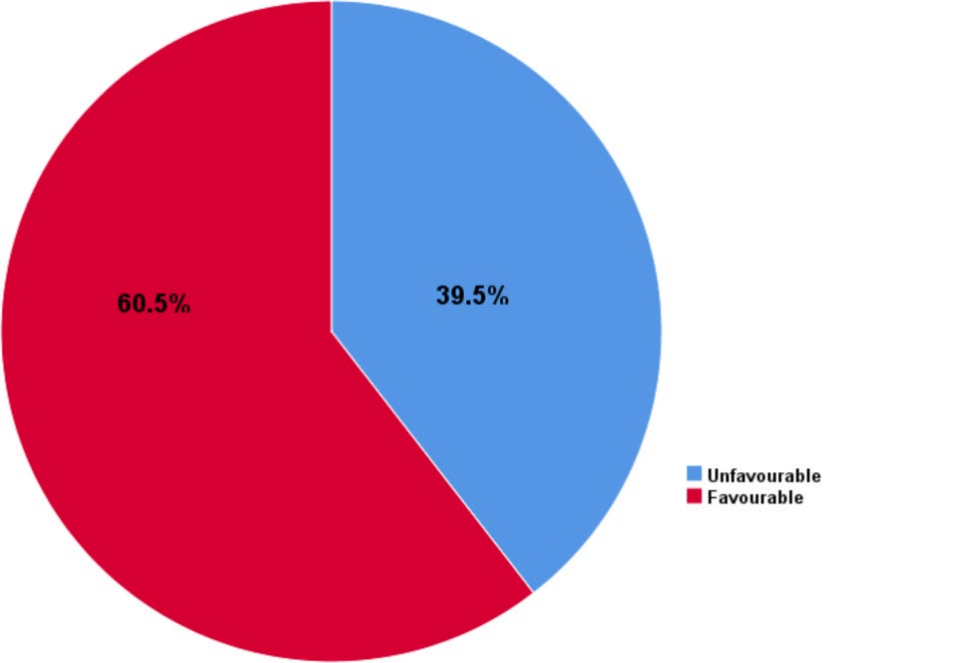
The level of attitude towards hepatitis B and C infection (*n* = 630)

According to this, 381**(**60.5%), 95% CI: (56.65–64.30) of the participants had favourable attitude (Fig. 2).

### Participants’ practice towards hepatitis B and C virus infection

This study found that 353(56.0%) of the participats didn’t receive Hepatitis vaccination. 352(55.9%) of them didn’t ever screened for Hepatitis infection. Whereas, 417(66.2%) of them didn’t exchange intravenous drug use (Table 5).

**Table 5:** Practice of the participants towards hepatitis B and C virus infection (*n* = 630)

Practice towards Hepatitis infection	Response			
	Yes		No	
	Frequency(N)	Percent(%)	Frequency(N)	Percent(%)
Have you received hepatitis vaccination?	277	44.0	353	56.0
Have you ever been screened for hepatitis infection?	278	44.1	352	55.9
Have you exchange intravenous drug use?	213	33.8	417	66.2
Do you avoid meeting with hepatitis infected patients?	313	49.7	317	50.3
Do you ask your barber/tattooist to change blade/or for safe Equipment’s for ear and nose piercing or tattooing?	351	55.7	279	44.3

### The level of practice towards hepatitis B and C virus infection

The overall level of practice was computed from the score for practice related questions and the score of mean and above were considered as there is a good practice and the score of below mean were considered as there is poor practice. The mean score was obtained from the total sum score of correct/good response for total practice–related questions.

**Fig. 3:**
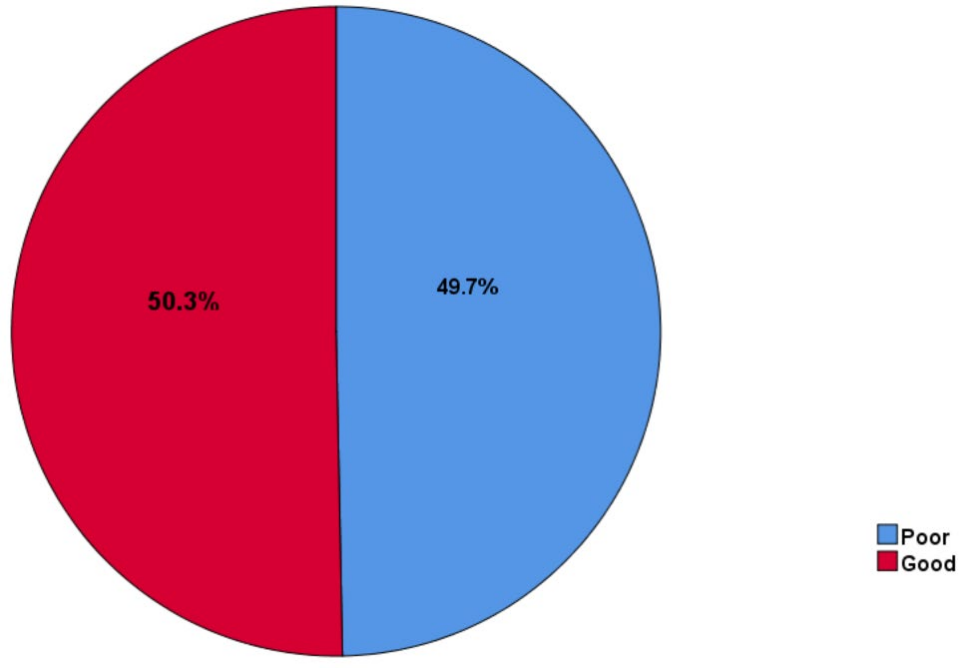
The level of practice towards hepatitis B and C infection (*n* = 630)

According to this, 317**(**50.3%), 95% CI: (46.40–54.23) of the participants had good practice (Fig. 3).

### Factors associated with knowledge towards hepatitis B and C virus infection

In bivariable analyses, knowledge towards Hepatitis B and C virus infection was significantly associated with sex, age, residence, marital status, educational status, occupational status, monthly income (in ETB), multiple sexual partner status, sharing sharp material, and vaccination status.

In multivariable analyses, from the variables which showed significant association in bivariable analyses, sex, number of sexual partner, sharing sharp material, and vaccination status were identified to be significantly associated with knowledge towards Hepatitis B and C infection.

According to this study, the odds of having good knowledge were 1.6 times [AOR = 1.61, 95%CI: (1.11–2.34)] higher among male individuals when compared to the female participants. Participants who had no multiple sexual partner found to have 0.5 times [AOR = 0.50, 95%CI: (0.25–0.98)] higher odds of having good knowledge when compared to the participants who had multiple sexual partner.

Participants who reported they didn’t share sharp materials with others were known to have 3.4 times [AOR = 3.35, 95%CI: (2.26–4.97)] more odds of having good knowledge when compared to their counterpart. In this study, the participants who were vaccinated for Hepatitis were known to have 1.98 times [AOR = 1.97, 95%CI: (1.26–3.10)] more odds to have good knowledge when compared to their counterpart (Table 6).

### Factors associated with attitude towards hepatitis B and C virus infection

In bivariable analyses, attitude towards Hepatitis B and C virus infection was significantly associated with sex, age, residence, marital status, educational status, occupational status, monthly income (in ETB), multiple sexual partner status, sharing sharp material, a tattooing history, and vaccination status.

In multivariable analyses, from the variables which showed significant association in bivariable analyses, residence, occupational status, income level, share sharp materials, and vaccination status were identified to be significantly associated with attitude towards Hepatitis B and C infection.

According to this study, the odds of having favourable attitude were 2 times [AOR = 2.10, 95%CI: (1.13–3.91)] higher among individuals who were dwelling in the urban area when compared to the individuals who were living in the rural area. Participants who a government employee were found to have 3.2 times [AOR = 3.21, 95%CI: (1.19–8.4)] higher odds of having favourable attitude when compared to the participants who were a farmers. This study also found that the participants who had monthly income level of > 4000 were revealed to have 2.8 times [AOR = 2.75, 95%CI: (1.00–7.52)] more odds of having favourable attitude when compared to the participants who had monthly income level of < 2000.

Participants who reported they didn’t share sharp materials with others were known to have 1.8 times [AOR = 1.76, 95%CI: (1.20–2.58)] more odds of having good knowledge when compared to their counterpart. In this study, the participants who were vaccinated for Hepatitis were known to have 1.8 times [AOR = 1.79, 95%CI: (1.14–2.82)] more odds to have good knowledge when compared to their counterpart (Table 6).

### Factors associated with practice towards hepatitis B and C virus infection

In bivariable analyses, practice towards Hepatitis B and C virus infection was significantly associated with sex, age, residence, marital status, educational status, occupational status, monthly income (in ETB), multiple sexual partner status, sharing sharp material, and vaccination status.

In multivariable analyses, from the variables which showed significant association in bivariable analyses, residence, occupational status, and vaccination status were identified to be significantly associated with practice towards Hepatitis B and C infection.

According to this study, the odds of having good practice were 2.2 times [AOR = 2.24, 95%CI: (1.25–4.02)] higher among individuals who were dwelling in the urban area when compared to the individuals who were living in the rural area. 4.15 (1.76–9.77 Participants who were the government employee have 4.15 times [AOR = 4.15, 95%CI: (1.76–9.77)], and merchants were 2.5 times [AOR = 2.47, 95%CI: (1.22–5.03)] higher odds of having good practice respectively when compared to the participants who were farmers. This study also found that the participants who were vaccinated for Hepatitis were revealed to have 4 times [AOR = 4.04, 95%CI: (2.57–6.35)] more odds of having good practice when compared to the participants who were not vaccinated for Hepatitis (Table 6).

**Table 6:** Multivariable analyses of factors associated with knowledge, attitude, and practice towards hepatitis B and C virus infection

Variable	Category	Knowledge		AOR(95% CI)	Attitude		AOR (95% CI)	Practice		AOR (95% CI)
		Good	Poor		Favorable	Unfavorable		Good	Poor	
Sex	Male	259	144	1.61(1.11–2.34)*	261	242	1.27(0.88–1.84)	226	177	1.31(0.90–1.91)
	Female	107	120	1.00	120	107	1.00	91	136	1.00
Age in years	18–29 years	105	117	1.00	107	115	1.00	92	130	1.00
	30–41 years	184	107	1.12(0.73–1.72)	195	96	1.34(0.88–2.04)	162	129	0.91(0.59–1.40)
	> 41 years	77	40	1.13(0.65–1.94)	79	38	1.23(0.73–2.10)	63	54	0.85(0.5–1.47)
Residence	Urban	61	21	1.59(0.88–2.87)	66	16	2.10(1.13–3.91)*	61	21	2.24(1.25–4.02)*
	Rural	305	243	1.00	315	233	1.00	256	292	1.00
Marital status	Single	106	53	1.00	227	179	1.00	91	68	1.00
	Married	222	184	0.94(0.60–1.47)	153	134	0.84(0.54–1.31)	190	216	0.95(0.61–1.48)
	Widowed/Divorced	38	27	1.00(0.51–1.96)	44	21	1.27(0.65–2.50)	36	29	1.03(0.53–2.01)
Educational status	No formal education	198	146	1.00	200	144	1.00	172	172	1.00
	Primary school	50	65	0.84(0.53–1.35)	53	62	0.92(0.58–1.45)	37	78	0.67(0.41–1.09)
	Secondary school	54	40	0.91(0.55–1.52)	65	29	1.62(0.96–2.72)	56	38	1.35(0.81–2.25)
	College and above	64	13	1.86(0.88–3.90)	63	11	1.61(0.78–3.34)	52	25	0.98(0.49–1.95)
Occupational status	Government employed	54	6	2.63(0.98–7.04)	54	6	3.21(1.19–8.64)*	50	10	4.15 (1.76–9.77) *
	Day laborer	21	19	0.96(0.48–1.92)	21	19	0.94(0.48–1.85)	16	24	0.85(0.42–1.72)
	Merchant	41	16	1.06(0.53–2.12)	42	15	1.22(0.60–2.47)	43	14	2.47(1.22–5.03)*
	Student	21	34	0.61(0.31–1.19)	24	31	0.76(0.40–1.45)	19	36	0.65(0.33–1.29)
	Farmer	229	189	1.00	240	178	1.00	189	229	1.00
Monthly income in ETB	< 2000	263	212	1.00	277	198	1.00	223	252	1.00
	2000–4000	64	44	0.93(0.58–1.49)	62	46	0.79(0.49–1.26)	57	51	1.09(0.67–1.77)
	> 4000	39	8	1.47(0.62–3.50)	42	5	2.75(1.00-7.52)*	37	10	2.05(0.90–4.66)
Multiple sexual partner	Yes	46	15	1.00	42	19	1.00	44	17	1.00
	No	320	249	0.50(0.25–0.98)*	339	230	1.04(0.55–1.98)	273	296	0.57(0.30–1.09)
Share sharp materials	Yes	65	121	1.00	86	100	1.00	85	101	1.00
	No	301	143	3.35(2.26–4.97)*	295	149	1.76(1.20–2.58)^*^	232	212	0.87(0.59–1.28)
Vaccination	Yes	115	40	1.97(1.26–3.10)*	117	38	1.79(1.14–2.82)^*^	121	34	4.04(2.57–6.35)*
	No	251	224	1.00	264	211	1.00	196	279	1.00

***Notes*** 1.00 = reference, * = Significant at: P-value ≤ 0.05, CI = Confidence Interval, AOR = Adjusted Odd Ratio

## Discussion

Based on the findings of this study, 366**(**58.1%), 95% CI: (54.23–61.96) of the participants had good knowledge. 381**(**60.5%), 95% CI: (56.65–64.30) of the participants had favourable attitude. and 317**(**50.3%), 95% CI: (46.40–54.23) of the participants had good practice. From factors analysis, sex, number of sexual partner, sharing sharp material, and vaccination status were identified to be significantly associated with good knowledge towards Hepatitis B and C virus infection. Residence, occupational status, monthly income level, sharing sharp material, and vaccination status were identified to be significantly associated with favourable attitude towards Hepatitis B and C virus infection. And residence, occupational status, and vaccination status were identified to be significantly associated with good practice towards Hepatitis B and C virus infection.

The magnitude of good knowledge indicated in this study is higher than the findings of previous studies conducted in Malaysia (37%) [9], in Cameron (36.0%) [25], and in Ethiopia (89.6%) [18]. The differences in socio economic status among settings, cultural behaviours, and also the time gap can contribute to this difference. However, it is lower than the result of previous studies conducted in Ethiopia: Woldia (52%); [15] Gonder(26.6-73.1%) [21, 23], and Jimma (73.9%) [22]. This relative decrease in the magnitude of good knowledge can be due to the time gap and population difference; they were health science students (at Woldia) and healthcare professionals (at Gonder). This may be because health science students and healthcare professionals can have better access to health-related information than non-health care professionals. Moreover, local contexts vary from place to place in Ethiopia.

The magnitude of favourable attitude found in this study is lower than the result of the study conducted in Khartoum, Sudan (86.4%) [26] and in Guangdong Province, China (83.3%) [16], and in Ethiopia, Jimma (88.7%) [22]. The possible reason for this difference may be due to the differences in population; they were healthcare providers. This may be because healthcare providers can have better access to hepatitis infection-related information than non-health care professionals and this can have the positive effect on their attitude level. And the time gap can contribute for this variation. Whereas it is higher than the findings of previous studies conducted in Cameron(54.6%) [25], and Ethiopia: Gonder (53.3%) [18]. The possible reason for this difference may be due to the differences in socio economic status among settings, time gap, and population behaviour difference from place to place also can contribute to this variation.

The magnitude of good practice revealed in this study is lower than the result of the study conducted in in Khartoum, in Sudan (65.5%) [26]. Whereas it is higher than the findings of previous studies conducted in Cameron(24.3%) [25]. The possible reason for this difference can be the variations in socio economic status among settings and time gap can contribute to this difference. Also it is lower than the findings of the study conducted in Gambela where 98.5% of the participants were not vaccinated for hepatitis B. and 87.2% of the participants had never been screened for hepatitis B or C [13], The possible reason for this difference can be the differences in socio economic status among settings, the time gap, and population variation; they were refugees (at Gambella). This may be because refugee’s living condition and the environment can make them more prone to poor practice. And also cultural behaviours variation and time gap can contribute to this difference.

This study finding revealed that there were higher odds of having favourable attitude among respondents who had an average monthly income level of > 4000 when compared to the participants who had < 2000. This implies that increment in income level has a positive effect on the improvement of attitude level [23]. This is supported by the study conducted in Northwest Ethiopia. This found that the increment on income level has a significant positive effect on the attitude level.

This study found that the respondents who were vaccinated for hepatitis had higher odds of having good knowledge, good practice, and favourable attitude when compared to their counterpart. This implies that vaccination history has a positive effect on the practice level. This is in line with the findings of previous study conducted in Northeast [15] and Northwest [18] Ethiopia. That found those vaccinated participants had a higher odds of having good knowledge, favourable attitude, and good practice when compared to those participants who were not vaccinated.

The present study revealed the current image of knowledge, attitude, and practice towards hepatitis B and C infection from the community side. However, there is a possibility of interviewer introduced bias and there may be the possibility of over-reporting. But the effort was made to minimize it through a genuine explanation of the objectives and significance of the study, and by recruiting interviewer from another kebele other than their own kebele. Moreover, the findings of this study is prone to subjective bias due to purposive sampling and cannot be applied to other settings but can be applied only to the community level settings.

## Conclusion

According to the study findings, it could be observed that good knowledge, favourable attitude, and good practice were indicated nearly above the half, only by half, and nearly above the half of the study participants respectively. In this study, sex, number of sexual partner, sharing sharp material, and vaccination status were identified to be significantly associated with knowledge towards Hepatitis B and C infection; residence, occupational status, average monthly income level, sharing sharp materials, and vaccination status were identified to be significantly associated with attitude towards Hepatitis B and C infection, and residence, occupational status, and vaccination status were identified to be significantly associated with practice towards Hepatitis B and C infection.

Implementing actions that could increase the awareness regarding limiting the number of sexual partner, not sharing sharp materials and urbanization is recommended. Moreover, woreda administrators, and other related authorities should consider knowledge, attitude, and practice as an implementation area, and also it would be better to create an opportunities to promote vaccination practices.

### Electronic supplementary material

Below is the link to the electronic supplementary material.

Supplementary Material 1

## Data Availability

The data sets used/or analyzed during the current study are available from the corresponding author on reasonable request.

## Declarations

### Authors’ information

Tamiru Chonka (BScN, MSc. in Pediatric and Child Health Nursing), School of Nursing, Department of Comprehensive Nursing, College of Medicine and Health Sciences, Arba Minch University.

Gesila Endashaw (BScN, M.Sc. Asst. Prof. in Maternity and Reproductive Health Nursing), School of Nursing, Department of Comprehensive Nursing, College of Medicine and Health Sciences, Arba Minch University.

Eshetu Zerihun (MPH. Asst. Prof. in Nutrition), School of Public Health, Department of Nutrition, College of Medicine and Health Sciences, Arba Minch University.

Bereket Beyene (BScN, M.Sc. in Adult Health Nursing), School of Nursing, Department of Comprehensive Nursing, College of Medicine and Health Sciences, Arba Minch University.

## Abbreviations

CDC: Centers for Disease Control and Prevention
ECDC: European Centre for Disease Prevention and Control
HBV: Hepatitis B Virus
HCV: Hepatitis C Virus
MOH: Ministry of Health
KAP: Knowledge, Attitude and Practice
NGOs: Non-Governmental Organizations
WHO: World Health Organization
