# Supplementary material for: Knowledge, attitude, and practice towards hepatitis B and C virus infection and associated factors among adults living at selected woredas in Gamo Zone, Southern Ethiopia: a cross-sectional study
Source: BMC Public Health. 2024 Apr 9;24:995. doi: 10.1186/s12889-024-18387-z (PMC11005261; doi:10.1186/s12889-024-18387-z)
Supplement: Supplementary file 1 — Supplementary Material 1 [file 12889_2024_18387_MOESM1_ESM.docx]

**STROBE Statement**

|  | Item No | Recommendation | Page number; line number reported |
| --- | --- | --- | --- |
| **Title and abstract** | 1 | (*a*) Indicate the study’s design with a commonly used term in the title or the abstract | 1 ; 3, 2 ; 29 |
|  |  | (*b*) Provide in the abstract an informative and balanced summary of what was done and what was found | 2 ; 30 – 49 |
| Introduction | | |  |
| Background/rationale | 2 | Explain the scientific background and rationale for the investigation being reported | 3 - 5 ; 51 – 118 |
| Objectives | 3 | State specific objectives, including any pre-specified hypotheses | 5 ; 116 – 118 |
| Methods | | |  |
| Study design | 4 | Present key elements of study design early in the paper | 6 ; 127 – 128 |
| Setting | 5 | Describe the setting, locations, and relevant dates, including periods of recruitment, exposure, follow-up, and data collection | 5 - 6 ; 120 – 126 |
| Participants | 6 | (*a*) Give the eligibility criteria, and the sources and methods of selection of participants | 6 - 7 ; 129 – 140 |
| Variables | 7 | Clearly define all outcomes, exposures, predictors, potential confounders, and effect modifiers. Give diagnostic criteria, if applicable | 8 ; 175 – 178 |
| Data sources/ measurement | 8* | For each variable of interest, give sources of data and details of methods of assessment (measurement). Describe comparability of assessment methods if there is more than one group | 8 - 9 ; 179 – 202 |
| Bias | 9 | Describe any efforts to address potential sources of bias | N/A |
| Study size | 10 | Explain how the study size was arrived at | 6 - 7 ; 141 – 154 |
| Quantitative variables | 11 | Explain how quantitative variables were handled in the analyses. If applicable, describe which groupings were chosen and why | 9 ; 212 – 222 |
| Statistical methods | 12 | (*a*) Describe all statistical methods, including those used to control for confounding | 9 ; 212 – 222 |
|  |  | (*b*) Describe any methods used to examine subgroups and interactions | N/A |
|  |  | (*c*) Explain how missing data were addressed | N/A |
|  |  | (*d*) If applicable, describe analytical methods taking account of sampling strategy | N/A |
|  |  | (*e*) Describe any sensitivity analyses | N/A |
| Results | | |  |
| Participants | 13* | (a) Report numbers of individuals at each stage of study—eg numbers potentially eligible, examined for eligibility, confirmed eligible, included in the study, completing follow-up, and analyzed | 10 ; 225 – 226 |
|  |  | (b) Give reasons for non-participation at each stage | N/A |
|  |  | (c) Consider use of a flow diagram | N/A |
| Descriptive data | 14* | (a) Give characteristics of study participants (eg demographic, clinical, social) and information on exposures and potential confounders | 10 - 11 ; 226 - 242 |
|  |  | (b) Indicate number of participants with missing data for each variable of interest | N/A |
| Outcome data | 15* | Report numbers of outcome events or summary measures | 12 - 16 ; 243 – 280 |
| Main results | 16 | (*a*) Give unadjusted estimates and, if applicable, confounder-adjusted estimates and their precision (eg, 95% confidence interval). Make clear which confounders were adjusted for and why they were included | 16 – 20 ; 281 – 342 |
|  |  | (*b*) Report category boundaries when continuous variables were categorized | N/A |
|  |  | (*c*) If relevant, consider translating estimates of relative risk into absolute risk for a meaningful time period | N/A |
| Other analyses | 17 | Report other analyses done—eg analyses of subgroups and interactions, and sensitivity analyses | N/A |
| Discussion | | |  |
| Key results | 18 | Summarize key results with reference to the study objectives | 21 ; 344 – 353 |
| Limitations | 19 | Discuss the limitations of the study, taking into account sources of potential bias or imprecision. Discuss both direction and magnitude of any potential bias | 24 ; 400 – 405 |
| Interpretation | 20 | Give a cautious overall interpretation of results considering objectives, limitations, multiplicity of analyses, results from similar studies, and other relevant evidence | 22 - 24; 354 - 400 |
| Generalizability | 21 | Discuss the generalizability (external validity) of the study results | 24 ; 405 |
| Other information | | |  |
| Funding | 22 | Give the source of funding and the role of the funders for the present study and, if applicable, for the original study on which the present article is based | Arba Minch University provided funds for the data collection and stationary materials of this research work with a project grant code: GOV/AMU/TH15/CMHS/NUR/04/14.  The website of the university is [www.amu.edu.et](http://www.amu.edu.et).  The funder had no role in study design, data collection, data analysis, data interpretation, writing of the final report, decision to publish or preparation of the manuscript. |
